# Saliva, a Magic Biofluid Available for Multilevel Assessment and a Mirror of General Health—A Systematic Review †

**DOI:** 10.3390/bios9010027

**Published:** 2019-02-14

**Authors:** Aranka Ilea, Vlad Andrei, Claudia Nicoleta Feurdean, Anida-Maria Băbțan, Nausica Bianca Petrescu, Radu Septimiu Câmpian, Adina Bianca Boșca, Bianca Ciui, Mihaela Tertiș, Robert Săndulescu, Cecilia Cristea

**Affiliations:** 1 Iuliu Haţieganu University of Medicine and Pharmacy Cluj-Napoca, Department of Oral Rehabilitation, Oral Health and Dental Office Management, Faculty of Dentistry, Cluj-Napoca 400012, Romania; anida.baezamica@yahoo.ro (A.-M.B.); nausica_petrescu@yahoo.com (N.B.P.); rcampian@email.com (R.S.C.); 2 DMD, Rezident doctor in Periodontology, Clinical County Hospital, Târgu Mureș 540136, Romania; 3 Iuliu Haţieganu University of Medicine and Pharmacy Cluj-Napoca, Department of Histology, Faculty of Medicine, Cluj 400349, Romania; 4 Iuliu Haţieganu University of Medicine and Pharmacy Cluj-Napoca, Department of Analytical Chemistry, Faculty of Pharmacy, Cluj 400349, Romania; ciuibianca@yahoo.com (B.C.); mihaela_tso@yahoo.com (M.T.); rsandulescu@umfcluj.ro (R.S.); ccristea@umfcluj.ro (C.C.)

**Keywords:** saliva, biofluid, biosensor, analytes, systematic review

## Abstract

Background: Saliva has been recently proposed as an alternative to classic biofluid analyses due to both availability and reliability regarding the evaluation of various biomarkers. Biosensors have been designed for the assessment of a wide spectrum of compounds, aiding in the screening, diagnosis, and monitoring of pathologies and treatment efficiency. This literature review aims to present the development in the biosensors research and their utility using salivary assessment. Methods: a comprehensive literature search has been conducted in the PubMed database, using the keywords “saliva” and “sensor”. A two-step paper selection algorithm was devised and applied. Results: The 49 papers selected for the present review focused on assessing the salivary biomarkers used in general diseases, oral pathologies, and pharmacology. The biosensors proved to be reliable tools for measuring the salivary levels of biochemical metabolic compounds such as glucose, proteinases and proteins, heavy metals and various chemical compounds, microorganisms, oncology markers, drugs, and neurotransmitters. Conclusions: Saliva is a biofluid with a significant clinical applicability for the evaluation and monitoring of a patient’s general health. Biosensors designed for assessing a wide range of salivary biomarkers are emerging as promising diagnostic or screening tools for improving the patients’ quality of life.

## 1. Introduction

Nowadays, human biological samples are used not only for the screening and diagnosis of various pathologies but also for assessing the compliance to treatment and the therapeutic efficacy. Depending on the types of investigations required, several options are available, varying from usual specimens (blood, plasma, saliva, sputum, urine, and feces) to more specific ones (cerebrospinal fluid, bone marrow, etc.). Blood collection is typically invasive and uncomfortable for the patients, being associated with high levels of anxiety, whereas urine or feces testing is considered to be privacy-invading [1,2,3]. 

In recent years, saliva has been explored as an alternative for the evaluation of homeostasis and the detection of pathologic conditions [4]. Most of the saliva (90%) is produced by the three major salivary glands (parotid, submandibular, and sublingual glands), whereas a small amount (10%) is produced by the minor salivary glands, distributed in the labial, buccal, lingual, and palatal areas of the oral mucosa [3,5]. Even though saliva is composed of 99% water, it also contains numerous constituents diffused from blood through para-cellular or trans-cellular pathways [5,6]. Saliva performs multiple functions, including digestion (the lubrication and binding of the alimentary bolus and the initiation of starch digestion), gustatory sensation (by solubilisation of dry food), protection (mechanical mobilization of alimentary debris), and antibacterial activity (lysis of the bacterial cell wall due to lysozyme) [4].

The oral cavity is host to a large number of commensal bacteria, known as the oral microbiome. Bacteria that can be found on the different surfaces in the oral cavity are organised in large communities that thrive and support one another, known as the biofilm. Inside the biofilm, the different species of bacteria communicate with one another, thus enabling the host colonisation, offering defence against competing bacteria, or adapting when changes are made in the environment around them. The pathologic effect of the oral microbiome occurs when the defensive mechanisms of the host are overtaken or impaired due to general pathologies, such as autoimmune diseases or diabetes [7,8]. In a healthy individual, a number of over 700 different bacterial species were identified, of which more than half have never been cultivated. The number of bacterial species and the composition of the microbiome may vary not only from one person to another but also in different areas of the oral cavity [9]. Moreover, the oral microbiome can undergo changes under certain conditions, such as during radiation therapy [10], or in the presence of dental prosthetic rehabilitations [11,12]. On the poorly maintained or unadapted removable prosthesis, an increased accumulation of bacteria has been identified [12]. In the case of fixed rehabilitations, the high accumulation of bacteria can be associated with the surface roughness or the material chosen for the prosthetic piece. The corrosion effect of saliva on the metallic crowns could increase the bacterial accumulation. The corrosive proccess was investigated in vitro by Uriciuc et al. using CoCrMo and CoCrW alloys immersed into artificial saliva. CoCr-based alloys with tungsten (W) content presented a linear and stable anticorrosion tendency and are more suitable for using as a single-casted alloy in prosthetics dental structures [11,13].

Given the modifications that may occur in certain pathologies, practitioners should consider setting a baseline of the salivary values of these patients. In this regard, saliva should be collected after a thorough professional cleaning of the oral cavity, carried out by a dental practitioner. The longitudinal study conducted by Esin et al. revealed a reduction of the microbial load (*Streptococcus mutans* and *Lactobacillus spp.*) three months after professional cleaning; this data sustains our suggestion regarding the salivary profiling of certain patients [14]. Furthermore, in order to reduce the alimentary influence, the samples should be collected *à jeun* before any food intake.

The use of human saliva for the diagnosis of different pathologies and for the monitoring of treatment outcomes is enabled by several advantages. Saliva collection is easy and does not require medical training; thus, it is feasible to both patients and practitioners. The sampling is fast and cost efficient, and saliva storage and shipping are easier than that of most biological samples. Moreover, unlike blood samples that may need additional media to prevent clotting, saliva is not susceptible to transformations. Finally, the contamination risk for medical personnel is lower, since no needles are involved [3,4].

Biosensors are defined as medical devices capable of detecting or measuring chemical or biological reactions by generating signals when in contact with an analyte [15]. Since their proof of concept in 1906, biosensors have become attractive to researchers and medical practitioners as alternatives to regular, expensive, and time-consuming investigations [6,15].

The aim of the present paper was to perform a comprehensive analysis of the existing literature on the ability of biosensors to detect various compounds in human saliva and on their reliability as an alternative to traditional laboratory investigations.

## 2. Materials and Method

A systematic review based on the PRISMA (Preferred Reporting Items for Systematic Reviews and Meta-Analyses) checklist was carried out. A search in the electronic data base PubMed was conducted using the association of the keywords “saliva” and “sensor”.

In the first step of the review, the titles and abstracts of the returned articles were analysed. The potentially eligible articles had to be published in the last 10 years, between the 1st of January 2009 and the 31st of December 2018. Furthermore, the papers had to focus on the detection of parameters in saliva using biosensors. Only human studies were included. Lastly, the articles had to be written in English.

In the second step of the review, the full text of the potentially eligible articles was analysed. The full text of the final papers had to be available for reading or purchasing. Detailed information about the sensors, their characteristics, and applicability to human saliva had to be reported. The accepted forms of papers were basic research, cross-sectional studies, or cohort studies. A basic research study is defined as a research conducted in the laboratory for the characterization and evaluation of a medical device. A cross-sectional study is an observational study conducted on a set number of subjects, evaluating the medical device at a specific point in time. All the final articles had to have the references listed and must have been cited at least once. The number of citations was determined using the Web of Science Core Collection search tool and, where the articles were not available, the Google Scholar search engine was employed.

## 3. Results

The initial search in the PubMed database using the keywords “saliva” and “sensors” returned a total of 242 results. Out of the total number, 46 had to be excluded for being published before the 1st of January 2009, bringing the number to 196 results. Another 85 papers were eliminated for not being human studies, and 2 other papers were not written in English. The titles and abstracts of the remaining 109 articles were further analysed, and 48 were excluded for not making a reference to the use of biosensors in saliva.

**Figure 1:**
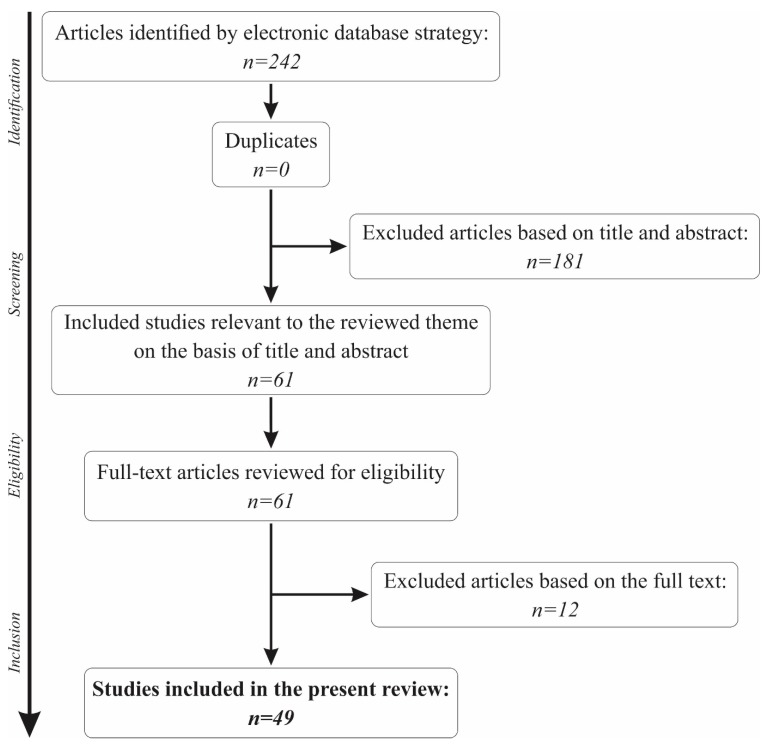
The paper selection algorithm for the present review.

A total of 61 articles remained for further analysis of the full text. Another 12 articles were removed from the present review for not making a reference to the main topic. Thus, a total of 49 papers were included in the present review. The full selection algorithm is visually represented in Figure 1.

Out of the total number of final articles, 4 were cross-sectional studies, whereas 45 were basic studies. Furthermore, 5 described the use of biosensors for the diagnosis of oral pathologies, 2 described their use in pharmacological monitoring, and 42 described their applicability in general pathologies screening, diagnosis, or follow-up.

**Figure 2:**
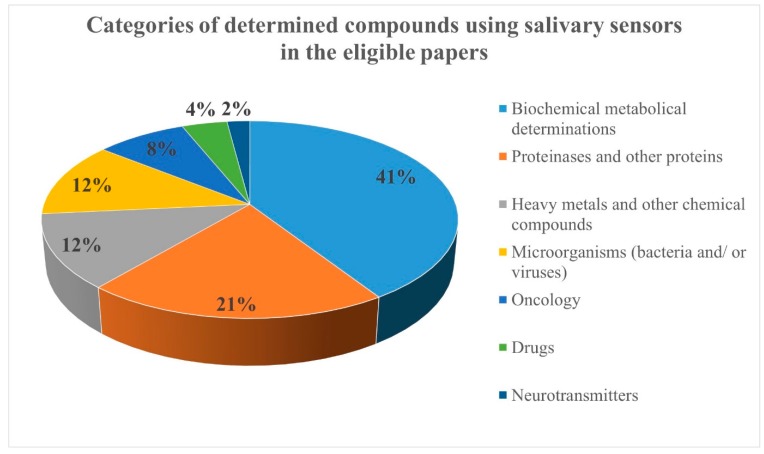
The categories of determined compounds using salivary sensors in the eligible papers.

Due to the large number of eligible articles, the compounds determined using salivary sensors were organized in seven categories: biochemical metabolic compounds (20 papers), proteinases and other proteins (10 papers), heavy metals and other chemical compounds (6 papers), microorganisms (bacteria and/or viruses; 6 papers), oncology markers (4 papers), drugs (2 papers), and neurotransmitters (1 paper) (Figure 2).

The final selected articles are listed in Table 1, with information on their authors, the original journal and year of publication, the analysed sensors and the assessed compounds, the purpose of determination, their indication, and the number of citations. These papers were sorted by the seven proposed categories and the year of publication. 

A comprehensive list containing the characterization information about each of the sensors from the final eligible papers, such as the limit of detection, selectivity, and sensitivity, alongside the author’s name, determined compounds, sensor information, and method of detection, can be found in Table 2.

## 4. Discussion

As revealed by the present literature review, the attention given to the applicability of biosensors for salivary determinations has increased in the recent years. The applications of these diagnostic tools have a wide spectrum, from general pathologies to dentistry and pharmacology.

### 4.1. Biochemical Metabolic Compounds

#### 4.1.1. Glucose

In the recent medical literature, there is a growing interest in metabolic products in saliva for monitoring the treatment effectiveness in various pathologies. The main focus was on the identification and quantification of glucose for monitoring diabetes. At the moment, the regular monitoring of blood glucose in diabetic patients involves the finger prick test, which is not only painful but also has been linked to major scarring in the fingers. Saliva, on the other hand, is easily collected without any of the aforementioned disadvantages. Furthermore, a strong correlation has been established between blood and the salivary levels of glucose in healthy patients, as well as in patients suffering from diabetes mellitus type 1 and 2. Thus, saliva could be an alternative biological fluid suitable for the monitoring of diabetes [2,63,64].

The use of sensors for detecting salivary glucose was reported in 2013 by Ye et al. who developed a CuO nanoneedle/graphene/carbon nanofiber modified glassy carbon electrode biosensor. The sensor was tested on saliva collected from healthy volunteers, and the findings indicated a rapid response as well as a high sensibility (minimum detection limit of 912.7 AmM^−1^ cm^−2^) and repeatability [16].

Other glucose-detecting sensors were developed by Li et al. in 2015 (electrochemical sensor using anodized cupric oxide nanowires, which was tested for calibration against serum glucose concentration), by Wang et al. in 2016 (core-shell IrO_2_@NiO nanowire), and by Du et al. in 2016 (a screen-printed sensor chip) [2,17,18].

A novel idea was the development of a constant-monitoring sensor designed for detecting surges of glucose intake in a patient over a set period of time. In that regard, Arakawa et al. proposed a sensor encased in a mouthguard wearable over a longer period of time. The mouthguard included a platinum and silver/silver chloride electrode, with glucose oxidase (GOD) immobilised by entrapment with Poly (MPC-co-EHMA) glucose sensor and a wireless transmitter. The mouthpiece was tested on a phantom jaw, using artificial saliva, a proved high sensitivity, and the ability to detect glucose in concentrations ranging from 5 to 1000 mmol/L [19].

Another personal-use device was developed by Soni et al. as a paper-based enzymatic sensor and a smartphone auxiliary device in order to reduce the dependability of expensive auxiliary devices for glucose determination in saliva. The paper-based sensor was designed to change colour in contact with glucose, the saturation being directly proportional to the concentration of glucose in the sample. Afterwards, the sensor was scanned with a special RGB-analysing software through a smartphone camera. The system was tested on both healthy and diabetic subjects, and a strong correlation between the salivary and blood glucose was reported (0.44 in healthy subjects, 0.64 in prediabetic patients, and 0.94 in diabetic patients) [20].

Other biochemical sensors for glucose included spectrophotometric detection using a low-cost colorimeter (Dominguez et al. 2017) [21]; using a colloidal AgNPs/MoS2-based nonenzymatic glucose biosensor (Anderson et al. 2017) [22]; tested on both saliva and sweat with similar performances; using randomly oriented CuO nanowire networks (Bell et al. 2017) [23]; using CuO-modified screen-printed carbon electrodes (SPCE; Velmurugan et al. 2017) [24]; using molecularly imprinted polymer binding on a conducting polymer layer (Kim et al. 2017) [25]; tested on both saliva and blood, methylene blue, hydrazine and platinum nanoparticles (Dutta et al. 2017); and using paper-based sensors (Santana-Jiménez et al. 2018) [26,27], standing to prove a high interest in the development of these medical devices.

#### 4.1.2. Cortisol and Cortisone

Cortisol is known to be a steroid hormone correlated with the stress levels, as well as other pathologies, such as Cushing’s syndrome and Addison’s disease. A directly proportional correlation was established between the circadian variations of cortisol concentration in blood and saliva, which led to the development of sensors able to detect and quantify cortisol salivary levels [28,65,66].

The first biosensor able to detect cortisol was developed by Mitchell et al., as a surface plasmon resonance (SPR) immunosensor. After being tested on both a buffer solution and on human saliva from healthy volunteers the sensor was proved to be highly sensitive (lower detection limit of 162 RU.mL/ng) [28].

Another SPR immunosensor was used by Frasconi et al. for the detection of both cortisol and cortisone. The sensors had a low response time (15−20 min), a high reusability (up to 100 times), and a low detection limit (3 μg L^−1^). The sensor was tested on both saliva and urine, using the proposed method and a reference liquid chromatography/tandem mass spectrometry method. [31].

Two other biosensors were used for the detection of cortisone by Pires et al. in 2014 (a chemiluminescent organic-based immunosensor) and by Usha et al. in 2017 (a lossy mode resonance-based fiber optic) [29,30].

#### 4.1.3. Other Biochemical Metabolic Compounds

Ballesta Claver et al. used an electrochemiluminescent biosensor for the detection of blood lactate in critical-state patients for preventing heart attacks in patients with diabetes mellitus in sports medicine as well as for food analysis [32].

A screen-printing technology on a flexible polyethylene terephthalate substrate was reported by Kim et al. for the detection and quantification of uric acid. The sensor was included in a wearable mouthguard connected to a wireless device through Bluetooth for data collection. The sensor was reported to have high selectivity (detection of 350 μM of uric acid in a solution with relevant physiological interferents), sensitivity (1.08 μA/mM), and stability; moreover, the idea of extending this continuous monitoring to other metabolites or substances was iterated [33].

Lastly, an electrochemical biosensor mounted on a mouthguard used for the monitoring of an advanced glycation end product, N-Carboxymethyl-lysine, was proposed by Ciui et al. The sensor proved high selectivity and sensitivity in a phosphate buffer with a limit of detection of 166 ng/mL (equivalent to 0.81 μM) over a range of 0.5–2500 μg/mL (equivalent to 2.45 μM–12.24 mM) of N-Carboxymethyl-lysine. Thus, the sensor proved a good reproducibility and a good selectivity against interferences from normal salivary constituents within physiological values. The short timescale required for the measurements, a long storage stability, and the ease of use are important advantages of the new mouthguard sensor [34].

### 4.2. Proteinases And Other Proteins

Salivary proteins have a major role in the digestive function of saliva. The concentration and activity of salivary amylase, one of the main components involved in oral digestion, has been correlated with the oral cancer, tobacco use, and cardiovascular disease. Hence, over the last years there was an increasing interest in developing sensors for salivary amylase detection and quantification. Lee et al. developed a biosensor consisting of molecularly imprinted thin films that, after being tested on saliva from five healthy subjects, proved an accuracy between 93.89% and 95.52% [35]. Another nano-sensor used for salivary amylase was developed by Attia et al. The sensors were applied on different starch-containing foods and showed high sensitivity (detection limit of 5.7 × 10^−11^ mol/L^−1^) [36].

Matrix metalloproteinases (MMP) are endopeptidases known for their ability to physiologically or pathologically cleave the components of the connective tissues. These enzymes, when activated by bacterial pathogens, have been linked to the destruction of periodontal soft tissues in periodontitis; the increase in collagenases salivary levels has been demonstrated to occur before the structural damage, allowing for an early diagnosis [37,38]. In this regard, a surface plasmon resonance immunosensor has been developed by Mohseni et al. in 2016 based on a carboxymethyldextran hydrogel sensor chip with immobilized monoclonal MMP-9 antibodies for the detection of MMP-9. The sensor was tested on healthy saliva with different protein concentrations (10−200 ng/mL), revealing that the final device had a detection limit of 8 pg/mL [36].

Another sensor for detecting MMP-1, MMP-8, and MMP-9 was proposed by Ritzer et al. as particle-bound protease cleavable linkers, delivered as a diagnostic chewing gum, used for the early detection of peri-implantitis. The sensor was designed to cleave in the presence of MMPs, releasing a strongly bitter taste. The device was tested on 33 patients (14 healthy and 19 patients with signs of mucositis/peri-implantitis), and the reaction was significantly different in the two groups after both a 5−10 min. incubation (1.5 ± 0.8% versus 4.2 ± 3.34%) and a 60 min. incubation (7.6 ± 4.4% versus 17.1 ± 11.1%) [38].

Human serum albumin (HSA) is the most abundant protein in the human body, accounting for approximately 60% of the total plasma proteins. Under pathological conditions, such as stomatitis associated with chemotherapy or type 2 diabetes, the salivary concentrations of HSA rise above the normal 0.5 g/L [39,67]. Hence, the direct detection of HSA concentration in saliva using a homogeneous fluorescent sensor has been proposed by Rongsheng et al. This device was tested on saliva samples from healthy volunteers, aged between 18 and 65 years, and a detection time between 40 and 50 min. was reported. Furthermore, no cross-reactivity was observed against other plasma proteins, such as human insulin, human C-reactive protein (CRP), or human IgG [31].

Cystatins are a family of proteins implied in regulatory and protective processes in the body. Their quantification in human blood and urine was used for the diagnosis of several diseases (cancer, kidney failure), whereas a low concentration in human saliva (normal values between 0.36 and 4.8 μg/mL) may indicate a predisposition to caries or the presence of active carious processes [40,68]. Gorodkiewicz et al. proposed a surface plasmon resonance imaging (SPRI) biosensor for the detection of cystatin in blood, saliva, and urine. The sensor was tested on six saliva samples, alongside blood plasma and urine, and detected the protein in all samples with concentrations within the normal physiological limits [32].

Gorodkiewicz et al. also proposed a second SPRI immunosensor for the detection of cathepsin D (CatD) and cathepsin E (CatE), as well as a third sensor for the detection of cathepsin G (CatG) [41,42]. CatD and CatE are aspartic peptidases, and their increased concentrations are a prognostic marker of cancer. The selectivity of the SPRI immunosensor was tested against cathepsin B (CatB); the sensor was not influenced by the presence of CatB, even if the concentration was increased 1000-fold [33].

CatG plays a role in the early immune response, as well as in coagulation and normal tissue degradation. An increased activity of CatG was associated with obstructive pulmonary disease, cancer, or emphysema [42,69]. The developed SPRI immunosensor used a specifically synthesised CatG inhibitor, MARS-115, which showed no response to other cathepsins. The sensor was tested for six saliva samples and accurately identified and quantified the peptidase [34].

An electrochemical sensor for the early detection of oral cancer was proposed by Wei et al., focusing on the salivary biomarkers interleukin (IL)-8 mRNA and IL-8 protein. After being tested on both oral cancer patients and healthy subjects, good specificity was proven through amperometric detection (IL-8 mRNA: −904 nA versus S100A8: −103 nA current; IL-8 protein: −298 nA versus IL-1 h protein: −50 nA) [43].

Lastly, the detection of L-tryptophan, a standard amino acid, was reported by Majidi et al., using two screen printed electrodes modified with a multiwall carbon nanotube (MWCNT-AuSPE) and then armed with Trp aptamer molecules (Apt-MWCNT-AuSPE). The sensors were tested against interferants (amino acids, glucose, and heavy metal ions), but the sensors produced no overlapping signals, even if the physiological concentrations exceeded by twenty-five fold [44].

### 4.3. Miscellaneous Chemical Compounds

The detection and monitoring of potassium iodine (KI) in saliva using a calixarene-based tubes ISFET (ion-selective field effect transistor) system was proposed by Puchnin et al., considering the high concentration of the drug in the salivary glands. The ISFET sensor modified with self-assembly Calixtube Monolayers allows the formation of inclusion complexes with KI, proving the specific discrimination between KI and other iodides due to the specific dimension of the molecule. The sensor detects the KI not the cation or the anion. KI is used for the treatment of dermatological inflammatory diseases. The sensor was tested for selectivity in artificial saliva, both spiked with KI and KI-free, proving distinctive responses in both situations. The detection limit was approximately 3 × 10^−8^ M, showing promises for clinical applications [45].

Minami et al. developed a nitrate biosensor based on the extended-gate type organic field-effect transistor. Nitrate can be found as an additive in processed food; it is also used for the prevention of cardiovascular disease. A high intake of this substance can cause different forms of cancer [46,70]. The sensor was applied on diluted human saliva, obtained from a healthy volunteer, in order to test its specificity. The recovery for the added nitrate solution was estimated at 97.4 ± 1.8%, comparable to other commercially available colorimetric-based devices [38].

A potentiometric membrane sensor was designed by Hassan et al. for the detection of thiocyanate, a compound excreted in urine and saliva and considered a biomarker for smokers and non-smokers. The sensor was tested on saliva and urine samples from both smokers and non-smokers and proved low detection limits (5.6 × 10^−6^ mol/L) and a rapid response time (10 s) [47].

Another sensor was developed for the detection of silver and mercury by Zheng et al. The sandwich-structured SERS probe with a gold nanohole array pattern proved a limit of detection for silver of 0.17 nM and a limit of detection for mercury of 2.3 pM [1].

Caffeine was identified in human saliva for the purpose of monitoring the drug metabolizing system activity in hepatocytes by Timofeeva et al. using a PVC membrane electrode biosensor. The device was tested on saliva provided by volunteers before and four hours after the ingestion of a caffeine pill, and it was proved that the normal metabolites in saliva did not interfere with the detection process, even in excess of 100- to 200-fold. Moreover, the sensor was tested using the already established HPLC (high performance liquid chromatography) method, showing insignificant differences between the two methods at a 95% confidence level [48].

Lastly, Zilberman et al. reported the development of a portable optoelectronic microfluidic sensor used for the detection of ammonia and carbon dioxide in saliva, secreted by *Helicobacter pylori*, used in the screening of stomach cancer. The sensor was tested on both healthy, unaltered saliva, as well as on saliva spiked with carbon dioxide and ammonia, showing good sensibility and selectivity [49].

### 4.4. Bacteria and Viruses

The direct determination of pathogens, such as bacteria and viruses, is of great importance not only in the early diagnosis of diseases but also in the monitoring the treatment efficiency.

Ahmed et al. proposed an impedimetric biosensor for the detection of a group A *Streptococcus*, *S. pyogenes*, incriminated for causing suppurative infections and possibly leading to streptococcal shock-like syndrome. The sensor’s properties were tested on human saliva, spiked with *S. pyogenes* (10^7^ cells/mL), and the bacteria showed a 4% charge transfer resistance, proving a high selectivity [50].

A microfluidic system (SLIM) for the detection of both bacteria and viruses was developed by Jin et al. The system was tested on 10 saliva samples that were previously positively diagnosed with a herpes zoster infection by real-time PCR. All of the samples were confirmed using the SLIM system and showed a sensitivity of 78.6% [54].

Xue et al. used an immunoassay based on microchannels within the multicapillary glass plate for the detection of viral antibodies and the diagnosis of viral infections. Recovery trials were conducted by adding standard h-IgA to six saliva samples collected from healthy volunteers and revealed that the system had a recovery ratio between 93.7%–112.2%, proving its high sensibility. Similar results were obtained by applying the system to environmental swabs and blood plasma [53].

Zaitouna et al. used an electrochemical peptide-based sensor enhanced with extra amino acids for the detection of anti-HIV antibodies. The peptide sensor spiked with amino acids had a selectivity factor of 7.8 and a limit of detection of 1 nM [55].

The detection of pathogens is also relevant in the early diagnosis of inflammatory periodontal diseases in both natural teeth and implants. *Porphyromonas gingivalis* proteases were determined by Wignarajah et al. using a multiplex colorimetric biosensor for detecting the chronic periodontal disease. The lower detection limit was reported at 1 pg/mL for HNE (Human Neutrophil Elastase) and 100 fg/mL for Cathepsin G, and the detection speed ranged between 20 and 30 s. [51].

Lastly, Hoyos-Nogués et al. used a peptide-based biosensor for the detection of *Streptococcus sanguinis*, a pathogen linked to inflammatory diseases involving dental implants. The sensor was tested in artificial saliva with varying concentrations of *S. sanguinis*, (10 to 10^5^ CFU·mL^−1^); the limit of detection was 8.6·10^2^ CFU·mL^−1^, with a sensitivity of 3.85 ± 1.3 kΩ per bacteria concentration decade [52].

### 4.5. Oncology Markers

The use of salivary biosensors also has applications in oncology, helping for the screening and early diagnosis of different cancers, by the detection of biomarkers. In this regard, Song et al. reported the development of a fluorescence-based immunosensor comprised of a hierarchical three-dimensional network of carbon nanotubes on a Si pillar substrate (3DN-CNTs). The sensor was used for the detection of a Cytokeratin-19 antigen (Cyfra 21-1) for the accurate diagnosis of oral squamous cell carcinoma (OSCC). The testing was carried out on 11 saliva samples (4 healthy and 7 from patients with OSCC), resulting in a limit of detection of 0.5 ng/mL and proving to be valid for clinical samples between 1 and 62.5 ng/mL [56].

Another fluorescent biosensor was designed by Chen et al., for the detection of the c-erbB-2 oncogene that could be useful in the early diagnosis of breast cancer. The device was tested for unstimulated saliva samples, with a detection limit of the oncogene of 20 fM and a small discrimination factor (approximately 1), proving its high specificity [57].

Cho et al. developed a surface-enhanced fluorescent optical sensor for the detection of the vascular endothelial growth factor-165 (VEGF165), a marker of cancer angiogenesis. The properties of the biosensor were tested on eight samples of stimulated saliva and blood plasma (four healthy and four with various forms of cancer), showing proportionate responses at VEGF165 concentrations from 25 pg/mL to 25 μg/mL [58].

Lastly, Yu et al. developed a capillary-based 3-D fluoro-immunosensor capable of detecting and quantifying the salivary levels of carcinoembryonic antigen which were involved in a wide array of cancers, with a limit of detection of 0.2 ng/mL and a relative mean recovery rate between 92.82% and 118.81% [59].

### 4.6. Drugs

The determination of drug intake using salivary biosensors could be useful for detecting illicit drugs intake. Machini et al. developed an electrochemical sensor based on a binuclear oxo-manganese complex for the detection of acetazolamide, a drug associated with doping in sports. The sensor testing in saliva samples revealed a detection limit of 4.76 × 10^−9^ mol·L^−1^ and maximum recovery errors of +2%, similar to the ones obtained in blood plasma and urine [60].

Biosensors and the determination of different compounds in saliva can also be applied in pharmacology for the evaluation of the efficiency of a treatment or the need for dosage adjustments. Yu et al. developed an electrochemical aptamer-based sensor for the detection of ampicillin in different biological fluids, including saliva. Ampicillin determination could be useful for determining the correct therapeutic concentration and the best way of administration. The sensor’s response was evaluated using two techniques: alternating current voltammetry (ACV) and square wave voltammetry (SWV), and the limit of detection varied from 1 μM (ACV) to 30 μM (SWV) [61].

### 4.7. Neurotransmitters

The detection of orexin A, an indicator used in the assessment of cognitive performance and fatigue, was accomplished by Hagen et al. using an electronic based (FET) biosensor. The compound was detected at concentrations of 10 fM in human saliva specimens and serum with the lowest detection limit established at sub-picomolar levels [62].

### 4.8. Future Perspectives

Considering the substantial data obtained through fundamental research on existing biosensors, several future perspectives can be drawn on these medical devices. Sensors need to be easily manufactured, at a low price, in order to be available to the wide population. Moreover, salivary biosensors need to be miniaturised and integrated in medical devices, such as mouthguards, or even included in dental prosthesis, dental restorations, or tooth surface retentions. However, this idea raises a few questions. Firstly, the mechanical retention of the device needs to be investigated and a correct long-term protocol for the adhesion of the sensor must be elaborated. Several steps may be necessary for the preparation of the teeth or prosthodontics pieces, such as the etching of the desired surfaces or the creation of micro-retentions using a sandblaster or burs. Secondly, the interference of food intake and microbiome with the readings must be controlled, as it may influence the data interpretation. Furthermore, the analysis of results has to exclude or take into account the oral pathology which can influence these dates recorded by salivary biosensor. Lastly, the durability and lifespan of the devices must be established in order for the medical personnel to plan the replacement of the sensor.

Wireless transmission of the data has already been achieved using Bluetooth connections. In theory, this could enable a continuous flow of the recorded information to already widespread and available mobile devices, allowing for a continuous monitoring by the patient or medical personnel. Such a large amount of data could be easily interpreted by doctors and alerts could be transmitted in real time as spikes in the normal readings. This would allow for the evaluation of drugs administration plans, the assessment of treatment efficiency, and the analysis of lifestyle.

Lastly, given the complex composition of saliva and its importance to the homeostasy of the oral cavity, further research might be needed in order to develop biosensors for all its components (Table 3) [71,72].

## 5. Conclusions

As shown in the present literature review, a great number of studies focused on the development of easy-to-use sensors with applicability on saliva, useful for general medicine, dental care, and pharmacology. As underlined by the authors, further clinical trials are required prior to the applicability of biosensors to the wide population. Furthermore, given the individual variations in salivary compounds, the need of setting a baseline for each patient should be analyzed.

Regarding the devices developed for long-term patient surveillance, such as mouthguards, clinical studies should investigate the potential toxic effects of the materials or of the sensors themselves. Even though biocompatible materials are being used by developers and researchers, further investigations should be conducted to prove that the device is suitable to be worn in the oral cavity.

As technology progresses, the high number of compounds that can be reliably detected in saliva is expected to increase. This could enhance the clinical applicability, provide reliable diagnostic or screening tools for the doctors, and improve the patients’ quality of life.

## Figures and Tables

**Table 1:** The final eligible papers for the present review.

No	Authors	Publication	Year	Type of Paper	Sensor	Determined Compounds	Purpose of Determination	Indication	Number of Times Cited
1	Ye et al. [16]	*Talanta*	2013	Basic research	CuO nanoneedle/graphene/carbon nanofiber modified glassy carbon electrode	Glucose	Diagnosis/Monitoring of diabetes mellitus	General pathologies	59
2	Li et al. [17]	*Sci Rep*	2015	Basic research	Electrochemical sensor using anodized cupric oxide nanowires	Glucose	Diagnosis/Monitoring of diabetes mellitus	General pathologies	36
3	Wang et al. [18]	*Anal Chem*	2016	Basic research	Core-shell IrO_2_@NiO nanowires	Glucose	Diagnosis/Monitoring of diabetes mellitus	General pathologies	16
4	Du et al. [2]	*J Diabetes Sci Technol*	2016	Basic research	Screen-printed sensor chip	Glucose	Diagnosis/Monitoring of diabetes mellitus	General pathologies	10 *
5	Arakawa et al. [19]	*Biosens Bioelectron*	2016	Basic research	Mouthguard glucose sensor	Glucose	Diagnosis/Monitoring of diabetes mellitus	General pathologies	31
6	Soni et al. [20]	*Anal Chim Acta*	2017	Cross-sectional study	Paper based sensor and smartphone RGB analysis	Glucose	Diagnosis/Monitoring of diabetes mellitus	General pathologies	2
7	Dominguez et al. [21]	*Sensors (Basel)*	2017	Cross-sectional study	Spectrophotometric detection	Glucose	Diagnosis/Monitoring of diabetes mellitus	General pathologies	1
8	Anderson et al. [22]	*Sensors (Basel)*	2017	Basic research	Colloidal AgNPs/MoS2-based nonenzymatic glucose biosensor	Glucose	Diagnosis/Monitoring of diabetes mellitus	General pathologies	4
9	Bell et al. [23]	*Nanotechnology*	2017	Basic research	Randomly oriented CuO nanowire networks	Glucose	Diagnosis/Monitoring of diabetes mellitus	General pathologies	4
10	Velmurugan et al. [24]	*J Colloid Interface Sci*	2017	Basic research	CuO modified screen printed carbon electrode (SPCE)	Glucose	Diagnosis/Monitoring of diabetes mellitus	General pathologies	10
11	Kim et al. [25]	*Biosens Bioelectron*	2017	Basic research	Molecularly imprinted polymer binding on a conducting polymer layer	Glucose	Diagnosis/Monitoring of diabetes mellitus	General pathologies	22
12	Dutta et al. [26]	*Biosens Bioelectron*	2017	Basic research	Methylene blue, hydrazine and platinum nanoparticles	Glucose	Diagnosis/Monitoring of diabetes mellitus	General pathologies	20
13	Santana-Jiménez et al. [27]	*Sensors (Basel)*	2018	Basic research	Paper-based sensors	Glucose	Diagnosis/Monitoring of diabetes mellitus	General pathologies	2
14	Mitchell et al. [28]	*Analyst*	2009	Basic research	Surface plasmon resonance (SPR) immunosensor	Cortisol	Detection and quantification of cortisol	General pathologies	35
15	Pires et al. [29]	*Biomed Mater Eng*	2014	Basic research	Chemiluminescent organic-based immunosensor	Cortisol	Detection and quantification of cortisol	General pathologies	6
16	Usha et al. [30]	*Biosens Bioelectron*	2017	Basic research	Lossy mode resonance-based fiber optic	Cortisol	Detection and quantification of cortisol	General pathologies	11
17	Frasconi et al. [31]	Anal Bioanal Chem	2009	Basic research	Surface plasmon resonance (SPR) immunosensor	Cortisol and cortisone	Detection and quantification of cortisol and cortisone	General pathologies	27
18	Ballesta Claver et al. [32]	*Analyst*	2009	Basic research	Electrochemiluminescent biosensor	Blood lactate	Detection and quantification of blood lactate	General pathologies	41
19	Kim et al. [33]	*Biosens Bioelectron*	2015	Basic research	Screen-printing technology on a flexible polyethylene terephthalate substrate	Uric acid	Detection and quantification of uric acid	General pathologies	70
20	Ciui et al. [34]	*Sens Actuators B Chem* (in press)	2019	Basic research	*Cavitas* electrochemical sensor in mouthguard	N-epsilon (carboxymethyl)lysine (CML)	Monitoring of CML	General pathologies	1 *
21	Lee et al. [35]	*ACS Appl Mater Interfaces*	2011	Basic research	Molecularly imprinted thin films	Salivary proteins	Detection and quantification of salivary proteins (a-amylase)	General pathologies	29
22	Attia et al. [36]	*Analyst*	2014	Basic research	Nano-optical sensor	Salivary proteins	Detection and quantification of salivary proteins (a-amylase)	General pathologies	22
23	Mohseni et al. [37]	*Biosens Bioelectron*	2016	Basic research	Carboxymethyldextran hydrogel sensor chip with immobilized monoclonal MMP-9 antibodies	Matrix metalloproteinases (MMP-9)	Diagnosis of chronic periodontal disease	Oral Pathologies	13
24	Ritzer et al. [38]	*Nat Commun*	2017	Basic research	Diagnostic chewing gum	Matrix metalloproteinases (MMP-1, MMP-8, MMP-9)	Diagnosis of inflammatory implant diseases	Oral Pathologies	8
25	Wang et al. [39]	*J Pharm Biomed Anal*	2012	Basic research	Homogeneous fluorescent sensor	Human serum albumin	Detection and quantification of human serum albumin	General pathologies	19
26	Gorodkiewicz et al. [40]	*Folia Histochem Cytobiol*	2012	Basic research	Surface Plasmon Resonance Imaging (SPRI) biosensor	Cystatin	Detection and quantification of cystatin	Oral pathologies	2
27	Gorodkiewicz et al. [41]	*Protein Pept Lett*	2010	Basic research	Surface plasmon resonance imaging (SPRI) biosensor	Cathepsin D (CatD) and cathepsin E (CatE)	Monitoring of cathepsin D and cathepsin E activity	General pathologies	16
28	Gorodkiewicz et al. [42]	*Anal Biochem*	2012	Basic research	Surface plasmon resonance imaging (SPRI) biosensor	Cathepsin G	Monitoring of Cathepsin G activity	General pathologies	21
29	Wei et al. [43]	*Clin Cancer Res*	2009	Basic research	Electrochemical (EC) sensor	IL-8 mRNA and IL-8 protein	Oncology (early cancer diagnostic)	General pathologies	111
30	Majidi et al. [44]	*Talanta*	2016	Basic research	Two ultrasensitive electrochemical sensor and aptasensor	Tryptophan	Selective analysis of tryptophan in biological samples	General pathologies	15
31	Puchnin et al. [45]	*Biosens Bioelectron*	2017	Basic research	Calixarene tubes	Potassium iodine (KI)	Detection and Monitoring of KI	General pathologies	3
32	Minami et al. [46]	*Biosens Bioelectron*	2016	Basic research	Organic field-effect transistors	Nitrate	Detection and quantification of nitrate ions	General pathologies	20
33	Hassan et al. [47]	*Anal Sci*	2009	Basic research	Potentiometric membrane sensor	Thiocyanate	Detection and quantification of thiocyanate	General pathologies	10
34	Zheng et al. [1]	*Nanoscale*	2015	Basic research	Sandwich-structured SERS probe with a gold nanohole array pattern	Silver and mercury	Detection of heavy metals intoxication	General pathologies	36
35	Timofeeva et al. [48]	*Talanta*	2016	Basic research	PVC membrane electrode	Caffeine	Monitoring of drug metabolizing system activity in hepatocytes	General pathologies	15
36	Zilberman et al. [49]	*Biosens Bioelectron*	2015	Basic research	Portable optoelectronic microfluidic sensor	Ammonia and carbon dioxide	Oncology (screening of stomach cancer)	General pathologies	18
37	Ahmed et al. [50]	*Anal Chem*	2013	Basic research	Impedimetric sensors	Pathogenic microorganisms (*Streptococcus pyogenes*)	Diagnosis of *Streptococcus pyogenes* infections	General pathologies	46 *
38	Wignarajah et al. [51]	*Anal Chem*	2015	Basic research	Multiplex colorimetric biosensor	Pathogenic microorganisms *(Porphyromonas gingivalis* proteases)	Diagnosis of chronic periodontal disease	Oral Pathologies	18
39	Hoyos-Nogués et al. [52]	*Biosens Bioelectron*	2016	Basic research	Peptide-based biosensor (hLf1-11)	Pathogenic microorganisms (*Streptococcus sanguinis*)	Inflammatory implant diseases	Oral Pathologies	16
40	Xue et al. [53]	*Sensors (Basel)*	2014	Basic research	Immunoassay utilizing microchannels within a multicapillary glass plate	Pathogenic microorganisms (detection of viral antibodies)	Diagnosis of viral infections	General pathologies	8
41	Jin et al. [54]	*Biosens Bioelectron*	2018	Cross-sectional study	Microfluidic system (SLIM)	Pathogenic microorganisms (bacteria and viruses)	Ultrasensitive pathogen detection	General pathologies	1
42	Zaitouna et al. [55]	*Anal Chim Acta*	2015	Basic research	Electrochemical peptide based sensor enhanced with extra amino acids	Anti-HIV antibodies	Human Immunodeficiency Virus (HIV)	General pathologies	7
43	Song et al. [56]	*Anal Chim Acta*	2018	Cross-sectional study	3DN-CNTs sensor	Cyfra 21-1	Oncology (diagnosis of oral squamous cell carcinoma)	General pathologies	1
44	Chen et al. [57]	*Anal Chim Acta*	2014	Basic research	Fluorescent biosensor	c-erbB-2 oncogene tumor marker	Oncology (early breast cancer diagnostic)	General pathologies	18 *
45	Cho et al. [58]	*ACS Nano*	2012	Basic research	Surface-enhanced fluorescent optical sensor	Vascular endothelial growth factor-165 (VEGF165)	Oncology (early cancer diagnostic)	General pathologies	87 *
46	Yu et al. [59]	*Anal Chem*	2014	Basic research	Capillary-based 3D fluoroimmunosensor	Carcinoembryonic antigen	Oncology (early cancer diagnostic)	General pathologies	30
47	Machini et al. [60]	*Biosens Bioelectron*	2016	Basic research	Electrochemical sensor using binuclear oxo-manganese complex	Acetazolamide	Detection of doping-associated substances	Pharmacology	5
48	Yu et al. [61]	*Talanta*	2018	Basic research	Electrochemical aptamer-based sensor (E-AB)	Ampicillin	Determination of optimal therapeutic concentration and the most effective method of drug administration	Pharmacology	12
49	Hagen et al. [62]	*ACS Chem Neurosci*	2013	Basic research	Electronic based (FET) biosensor	Orexin A	Detection and quantification of orexin A	General pathologies	7

* Articles available only in the Google Scholar database.

**Table 2:** The characterization information on the sensors from the final eligible papers.

No	Authors	Determined Compounds	Sensor	Method of Detection	Limit of Detection	Selectivity	Sensitivity
1	Ye et al. [16]	Glucose	CuO nanoneedle/graphene/carbon nanofiber modified glassy carbon electrode	Amperometric detection	912.7 A·mM^−1·^cm^−2^	*N/A* ^†^	*N/A*
2	Li et al. [17]	Glucose	Electrochemical sensor using anodized cupric oxide nanowires	Electrochemical detection	0.3/μM	*N/A*	2217.4/μA·cm^−2^ mM^−1^
3	Wang et al. [18]	Glucose	Core-shell IrO_2_@NiO nanowires	Electrochemical detection	0.31 μM	*N/A*	1539.0 μA·mM^−1^·cm^−2^
4	Du et al. [2]	Glucose	Screen-printed sensor chip	Amperometric detection	1.1–45 mg/dL	*N/A*	*N/A*
5	Arakawa et al. [19]	Glucose	Mouthguard glucose sensor	Electrochemical detection	5 mmol/L	*N/A*	*N/A*
6	Soni et al. [20]	Glucose	Paper based sensor and smartphone RGB analysis	Colorimetric evaluation using an RGB sensor	24.6 mg/dL	*N/A*	0.0012 pixels s^−1^/mg·dL^−1^
7	Dominguez et al. [21]	Glucose	Spectrophotometric detection	Colorimetric evaluation using an RGB sensor	0.17 mg/dL	*N/A*	*N/A*
8	Anderson et al. [22]	Glucose	Colloidal AgNPs/MoS2-based nonenzymatic glucose biosensor	Electrochemical detection	0.03 μM	*N/A*	9044.6 μA·mM^−1^·cm^−2^
9	Bell et al. [23]	Glucose	Randomly oriented CuO nanowire networks	Amperometric detection	0.05 mM Glucose (Gl) at +0.6 V	*N/A*	0.1 nA/mM Gl in the 0–7 mM Gl range and 2.1 nA/mM Gl above 7 mM Gl
10	Velmurugan et al. [24]	Glucose	CuO modified screen printed carbon electrode (SPCE)	Electrochemical detection	0.1 μM	*N/A*	308.71 μA·mM^−1^ cm^−2^
11	Kim et al. [25]	Glucose	Molecularly imprinted polymer binding on a conducting polymer layer	Potentiometric measurements	1.9 (±0.15) × 10^−7^ M	*N/A*	*N/A*
12	Dutta et al. [26]	Glucose	Methylene blue, hydrazine and platinum nanoparticles	Oxidation current measurements	2.2 pg/mL	*N/A*	*N/A*
13	Santana-Jiménez et al. [27]	Glucose	Paper-based sensors	Naked-eye detection	47 μM	*N/A*	1.81 A.U./mg
14	Mitchell et al. [28]	Cortisol	Surface plasmon resonance (SPR) immunosensor	Surface plasmon resonance	49 pg/mL	*N/A*	162 RU.mL/ng
15	Pires et al. [29]	Cortisol	Chemiluminescent organic-based immunosensor	Organic photodetection	80 pg/mL	*N/A*	685 pg/mL
16	Usha et al. [30]	Cortisol	Lossy mode resonance-based fiber optic	Fiber optic real-time detection	25.9 fg/ml	*N/A*	*N/A*
17	Frasconi et al. [31]	Cortisol and cortisone	Surface plasmon resonance (SPR) immunosensor	Surface plasmon resonance	Cortisol: 4 μg·L^−1^ Cortisone: 10 μg·L^−1^	*N/A*	*N/A*
18	Ballesta Claver et al. [32]	Blood lactate	Electrochemiluminescent biosensor	Electrochemiluminescence detection	*N/A*	*N/A*	*N/A*
19	Kim et al. [33]	Uric acid	Screen-printing technology on a flexible polyethylene terephthalate substrate	Potentiometric measurements	*N/A*	350 μM	1.08 μA/mM
20	Ciui et al. [34]	N-epsilon (carboxymethyl)lysine (CML)	*Cavitas* electrochemical sensor in mouthguard	Electrochemical detection	0.81 μM	*N/A*	*N/A*
21	Lee et al. [35]	Salivary proteins	Molecularly imprinted thin films	Quartz crystal microbalance detection	0.1 mg/mL	*N/A*	*N/A*
22	Attia et al. [36]	Salivary proteins	Nano-optical sensor	Spectrofluorimetric detection	5.7 × 10^−1^ mol/L^−1^	*N/A*	*N/A*
23	Mohseni et al. [37]	Matrix metalloproteinases (MMP-9)	Carboxymethyldextran hydrogel sensor chip with immobilized monoclonal MMP-9 antibodies	Surface plasmon resonance	8 pg/mL	*N/A*	High (recovery rate of ~94%)
24	Ritzer et al. [38]	Matrix metalloproteinases (MMP-1, MMP-8, MMP-9)	Diagnostic chewing gum	Peptide sensors	*N/A*	*N/A*	*N/A*
25	Wang et al. [39]	Human serum albumin	Homogeneous fluorescent sensor	Fluorescence resonance energy transfer	3.9 ng/mL	*N/A*	*N/A*
26	Gorodkiewicz et al. [40]	Cystatin	Surface Plasmon Resonance Imaging (SPRI) biosensor	Surface Plasmon Resonance Imaging	0.1 μg/mL	*N/A*	*N/A*
27	Gorodkiewicz et al. [41]	Cathepsin D (CatD) and cathepsin E (CatE)	Surface plasmon resonance imaging (SPRI) biosensor	Surface Plasmon Resonance Imaging	0.12 ng mL^−1^	*N/A*	*N/A*
28	Gorodkiewicz et al. [42]	Cathepsin G	Surface plasmon resonance imaging (SPRI) biosensor	Surface Plasmon Resonance Imaging	0.23 ng/mL	*N/A*	High (recovery rate of 100%)
29	Wei et al. [43]	IL-8 mRNA and IL-8 protein	Electrochemical (EC) sensor	Electrochemical detection	IL-8 mRNA −3.9 fM and IL-8 protein: 7.4 pg/mL	~90%	~90%
30	Majidi et al. [44]	Tryptophan	Two ultrasensitive electrochemical sensor and aptasensor	Electrochemical detection and Electrochemical impedance spectroscopy	MWCNT-AuSPE: 3.6 × 10^−10^ mol L^−1^ and Apt-MWCNT-AuSPE: 4.9 × 10^−12^ mol L^−1^	*N/A*	*N/A*
31	Puchnin et al. [45]	Potassium iodine (KI)	Calixarene tubes	Ion-selective field effect detection	~3×10^−8^ M	*N/A*	*N/A*
32	Minami et al. [46]	Nitrate	Organic field-effect transistors	Organic field-effect detection	45 ppb	*N/A*	High (recovery rate of 97.4 ± 1.8%)
33	Hassan et al. [47]	Thiocyanate	Potentiometric membrane sensor	Potentiometric measurements	5.6 × 10^−6^ mol/L	*N/A*	−57.5 ± 0.5 mV decade^−1^
34	Zheng et al. [1]	Silver and mercury	Sandwich-structured SERS probe with a gold nanohole array pattern	Surface-enhanced Raman scattering detection	0.17 nM of Silver 2.3 pM of Mercury	*N/A*	*N/A*
35	Timofeeva et al. [48]	Caffeine	PVC membrane electrode	Flow potentiometric measurements	1.2 mg^−1^L	*N/A*	52 ± 1 mV dec^−1^
36	Zilberman et al. [49]	Ammonia and carbon dioxide	Portable optoelectronic microfluidic sensor	Optoelectronic detection	*N/A*		*N/A*
37	Ahmed et al. [50]	Pathogenic microorganisms (*Streptococcus pyogenes*)	Impedimetric sensors	Impedance-based electrochemical measurements	*N/A*	High (4% charge transfer resistance)	*N/A*
38	Wignarajah et al. [51]	Pathogenic microorganisms *(Porphyromonas gingivalis* proteases)	Multiplex colorimetric biosensor	Colorimetric detection	HNE: 1 pg/mL Cathepsin G: 100 fg/mL	*N/A*	*N/A*
39	Hoyos-Nogués et al. [52]	Pathogenic microorganisms (*Streptococcus sanguinis*)	Peptide-based biosensor (hLf1-11)	Electrochemical impedance spectroscopy	8.6 × 10^2^ CFU·mL^−1^	*N/A*	3.85 ± 1.3 kΩ per bacteria concentration decade
40	Xue et al. [53]	Pathogenic microorganisms (detection of viral antibodies)	Immunoassay utilizing microchannels within a multicapillary glass plate	Fluorescence detection	0.05 ng/mL	*N/A*	High (recovery ratio between 93.7%–112.2%)
41	Jin et al. [54]	Pathogenic microorganisms (bacteria and viruses)	Microfluidic system (SLIM)	Isothermal optical detection	*N/A*	*N/A*	78.6%
42	Zaitouna et al. [55]	Anti-HIV antibodies	Electrochemical peptide-based sensor enhanced with extra amino acids	Electrochemical detection	1 nM	*N/A*	Selectivity factor: 7.8
43	Song et al. [56]	Cyfra 21-1	3DN-CNTs sensor	Fluorescence detection	0.5 ng/mL	*N/A*	*N/A*
44	Chen et al. [57]	c-erbB-2 oncogene tumor marker	Fluorescent biosensor	Fluorescence detection	20 fM	High (discrimination factor ~ 1)	RSD = 1.46% (n = 8)
45	Cho et al. [58]	Vascular endothelial growth factor-165 (VEGF165)	Surface-enhanced fluorescent optical sensor	Fluorescence detection	25 pg/mL	*N/A*	*N/A*
46	Yu et al. [59]	Carcinoembryonic antigen	Capillary-based 3D fluoroimmunosensor	Fluorescence detection	0.2 ng/mL	*N/A*	High (recovery ratio between 92.82%–118.81)
47	Machini et al. [60]	Acetazolamide	Electrochemical sensor using binuclear oxo-manganese complex	Electrochemical detection	4.76 × 10^−9^ mol L^−1^	*N/A*	*N/A*
48	Yu et al. [61]	Ampicillin	Electrochemical aptamer-based sensor (E-AB)	Electrochemical aptamer detection	ACV: 1 μ MSWV: 30 μM	*N/A*	*N/A*
49	Hagen et al. [62]	Orexin A	Electronic based (FET) biosensor	Field-effect detection	sub-picomolar levels	*N/A*	*N/A*

^†^ N/A = non-applicable/not available information.

**Table 3:** The main salivary constituents and the biosensors developed for their determination.

Category	Compounds	Yes	No
**Electrolytes**	Sodium		x
	Potassium	x	
	Calcium		x
	Magnesium		x
	Phosphate		x
	Iodine	x	
	Chloride		x
	Bicarbonate		x
**Mucus**	Mucoplysaccharides		x
	Glycoproteins		x
**Antibacterial compounds**	Thiocyanate	x	
	Hydrogen peroxide		x
	Immunoglobulin A		x
	Immunoglobulin G		x
	Immunoglobulin M		x
	Limphocytes		x
	Monocites		x
**Enzymes**	α-amylase	x	
	Lipase		x
	Kallikrein		x
	Lysozyme		x
	Lactoperoxidase		x
	Lactoferrin		x
**Cells**	Desquamated epithelial cells		x
	Bacteria	x	
**Nitrogenous products**	Urea		x
	Uric acid	x	
	Ammonia	x	
**Amino acids**			
**Glucides**	Glucose	x	
**Epidermal growth factors**			x
**Proteins**		x	
